# Cardiovascular and Renal Outcomes Following Repeated Naringenin Exposure in Normotensive and Hypertensive Rats

**DOI:** 10.3390/ph18060873

**Published:** 2025-06-12

**Authors:** Anelize Dada, Rita de Cássia Vilhena da Silva, Mariana Zanovello, Anelise Felício Macarini, Thaise Boeing, Valdir Cechinel Filho, Priscila de Souza

**Affiliations:** Programa de Pós-Graduação em Ciências Farmacêuticas (PPGCF), Universidade do Vale do Itajaí (Univali), Itajaí 88302-901, Brazil

**Keywords:** naringenin, hypertension, renal function, urolithiasis, blood pressure, calcium oxalate

## Abstract

**Background**: Systemic arterial hypertension is one of the leading global health concerns, significantly increasing the risk of cardiovascular and kidney diseases, including nephrolithiasis. The treatment, still far from ideal, is constantly undergoing new alternatives. In this context, medicinal plants rich in flavonoids, such as naringenin—a compound found in citrus fruits—have gained attention for their potential diuretic, nephroprotective, and blood pressure-lowering effects. **Objectives**: This study aimed to evaluate the effects of naringenin (100 mg/kg, orally) over nine days on blood pressure, renal function, and calcium oxalate crystal formation in normotensive Wistar (NTR) and spontaneously hypertensive male rats (SHR). **Methods**: Key assessments included blood pressure and heart rate measurements in vivo, urine volume and electrolyte excretion in vivo, in vitro calcium oxalate crystallization, and in silico molecular docking analyses to investigate molecular interactions. **Results**: Naringenin treatment significantly reduced blood pressure and increased diuresis in both NTR and SHR groups, while a notable natriuretic effect was observed specifically in NTR. In vitro, naringenin reduced the formation of calcium oxalate crystals in urines from NTR. Molecular docking studies suggested that these effects may be mediated by interactions with SGLT1 and SGLT2 transporters, potentially explaining the diuretic and natriuretic outcomes. Additionally, interactions with MMP-9 and β2-adrenergic receptors may contribute to the reduction in crystal formation. **Conclusions**: Collectively, these findings indicate that repeated administration of naringenin exerts beneficial effects on both cardiovascular and renal parameters, and point to promising molecular targets that may underlie its protective actions.

## 1. Introduction

Systemic arterial hypertension (SAH) is recognized as one of the primary risk factors for cardiovascular morbidity and mortality worldwide, contributing to approximately 19% of all deaths globally. Despite its high prevalence and significant impact on public health, the precise etiology of SAH remains not fully elucidated. It is widely regarded as a multifactorial disease influenced by a complex interplay of genetic predisposition, environmental factors, neurohormonal dysregulation, renal dysfunction, and lifestyle-related aspects such as diet, physical inactivity, and stress [1,2]. It is one of the major cardiovascular risk factors and serves as a key contributor to the development of various severe health conditions, including atherosclerosis, acute myocardial infarction, heart failure, stroke, coronary artery disease, and chronic kidney disease. The persistent elevation of arterial blood pressure imposes mechanical stress on blood vessels and vital organs, leading to structural and functional damage over time. Consequently, uncontrolled hypertension significantly increases the risk of life-threatening cardiovascular and renal events, thereby reducing life expectancy and quality of life [3]. Hypertension is also closely associated with kidney dysfunction, given that the regulation of blood pressure is intricately managed through a combination of vascular mechanisms (such as vasodilation and vasoconstriction), neural pathways, and renal control systems. In particular, the kidneys play a pivotal role in maintaining blood pressure homeostasis via the renin–angiotensin–aldosterone system (RAAS) and other hormonal pathways that modulate fluid balance, sodium retention, and vascular resistance. Impairments in these systems not only contribute to the onset of hypertension but also accelerate kidney damage, creating a vicious cycle that exacerbates both conditions [4,5,6].

Nephrolithiasis is a significant public health concern that affects a substantial portion of the global population. It is characterized by the formation of kidney stones, which can lead to severe pain, urinary tract obstruction, and potential complications if left untreated. The development of kidney stones is multifactorial, influenced by genetic predisposition, dietary habits, inadequate fluid intake, and lifestyle factors. Moreover, nephrolithiasis is frequently associated with comorbid conditions such as obesity, diabetes, metabolic syndrome, and SAH, all of which contribute to alterations in urinary composition, promoting stone formation [7,8]. The most common crystals are those formed by calcium oxalate (CaOx), which can be monohydrated or dihydrated, differentiated by their morphology [9]. The increasing prevalence of this condition underscores the need for effective preventive and therapeutic strategies.

Several studies suggest the use of medicinal plants as an alternative treatment for nephrolithiasis, as well as for their diuretic effect and blood pressure control [10,11,12]. Within this class, flavonoids merit particular attention, with emphasis on naringenin—a flavanone commonly found in both its glycosylated form (naringin) and its aglycone form (naringenin). It is widely found in citrus fruits, particularly grapefruits, oranges, and tomatoes [13,14,15]. Studies demonstrate the important role of its effects on renal function, the ability to promote diuresis, prevention of kidney stone formation [16] and cardiovascular parameters [17]. Despite the extensive literature on the pharmacological properties of naringenin, significant gaps persist regarding its effects in experimental models of arterial hypertension, particularly in spontaneously hypertensive rats (SHR). This study hypothesizes that daily exposure to naringenin promotes renoprotection by modulating renal function and electrolyte balance, thereby contributing to blood pressure control in hypertensive conditions. Thus, the aim of this study was to evaluate the effect of naringenin on blood pressure and renal parameters in normotensive (NTR) and SHR groups, as well as to assess its protective effect on CaOx crystal formation in vitro.

As will be presented below, this manuscript provides novel insights by demonstrating, for the first time, the multifaceted effects of repeated naringenin administration on cardiovascular and renal functions in hypertensive and normotensive models. It elucidates its dual role as an antihypertensive and diuretic agent, along with its ability to reduce calcium oxalate crystal formation in vitro. Furthermore, the study identifies potential molecular targets involved in these effects, expanding the understanding of naringenin’s mechanisms of action and supporting its therapeutic potential in managing hypertension and kidney-related disorders.

## 2. Results

### 2.1. Evaluation of Blood Pressure Values in NTR and SHR Groups Treated or Not with Naringenin

**Figure 1:**
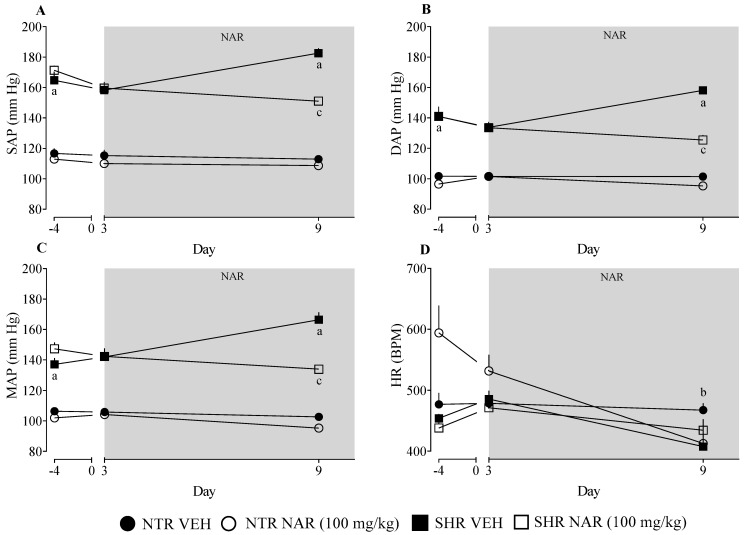
Assessment of blood pressure and heart rate in the experimental groups was performed using tail-cuff plethysmography. The parameters analyzed included the following: (**A**) systolic arterial pressure (SAP); (**B**) diastolic arterial pressure (DAP); (**C**) mean arterial pressure (MAP) in SHR; and (**D**) heart rate (HR). ^a^ *p* < 0.05 comparison between NTR vehicle and SHR vehicle. ^b^ *p* < 0.05 comparison between NTR vehicle and NTR NAR. ^c^ *p* < 0.05 comparison between SHR vehicle and SHR NAR.

To validate the difference in blood pressure between the NTR and SHR groups, blood pressure (BP) was measured before treatment began. Analysis of the results confirms a significant difference in systolic arterial pressure (SAP), diastolic arterial pressure (DAP), and mean arterial pressure (MAP) between the NTR and SHR groups (Figure 1A–C). However, no significant changes were observed in heart rate (HR) values (Figure 1D), with the exception of the NTR NAR group which was significantly reduced over the treatment period. In addition, naringenin, a flavonoid found in citrus fruits which, in previous studies, has shown positive effects in reducing blood pressure, was used to corroborate these data. BP measurements of the groups were taken on days 3 and 9 of treatment, and it was observed that, throughout the treatment, naringenin was able to reduce blood pressure levels in the SHR group compared to the vehicle (VEH)-only treated SHR group.

### 2.2. Evaluation of Urine Volume and pH

**Figure 2:**
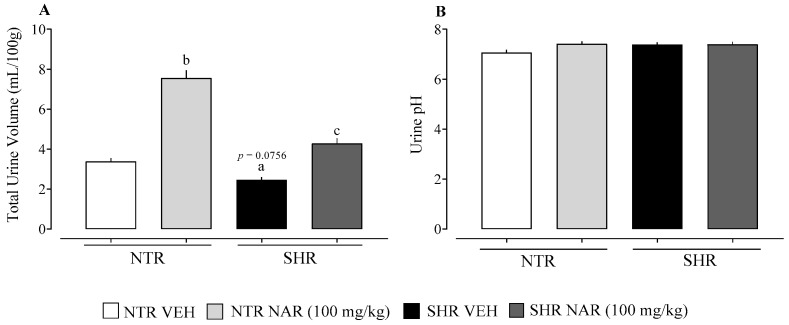
Cumulative effect of urine and pH of periodic treatment with naringenin in NTR and SHR groups. (**A**) Cumulative volume of urine; (**B**) cumulative urine pH. ^a^ *p* < 0.05 comparison between NTR and SHR vehicles. ^b^ *p* < 0.05, comparison between the NTR vehicle and NTR NAR. ^c^ *p* < 0.05, comparison between SHR vehicle and SHR NAR.

When assessing the volume of urine excreted by the groups, there was a tendency for the volume to decrease in the SHR VEH group compared to the NTR VEH group. In addition, a significant effect of naringenin was identified, which significantly increased urinary excretion in both the NTR and SHR groups. No significant differences were found in the analysis of urinary pH values (Figure 2).

### 2.3. Evaluation of Urinary Parameters

**Figure 3:**
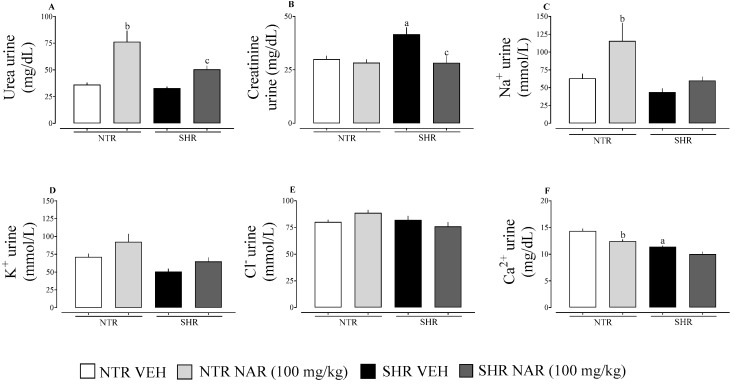
Urinary parameters in samples from the NTR and SHR experimental groups. (**A**) Urea; (**B**) creatinine; (**C**) Na^+^; (**D**) K^+^; (**E**) Cl^−^; (**F**) Ca^2+^. ^a^ *p* < 0.05 comparison between NTR and SHR vehicles. ^b^ *p* < 0.05, comparison between the NTR vehicle and NTR NAR. ^c^ *p* < 0.05, comparison between SHR vehicle and SHR NAR.

As shown in Figure 3, there were no significant differences between the groups in urinary K^+^ and Cl^−^ levels (panels D and E). When assessing Na^+^ levels in the urine, statistically higher levels were observed in the NTR NAR group, corroborating the findings regarding the volume of urine excreted, where this group showed greater excretion. In addition, an increase in urea levels was identified in the NAR-treated groups. Creatinine excretion was higher in the SHR VEH group. On the other hand, NAR was able to reduce this alteration in the SHR group. Calcium release through the urine was significantly lower in the SHR groups when compared to the NTR group. Furthermore, NAR reduced this excretion in the NTR group.

### 2.4. Evaluation of CaOx Crystal Formation in the Urine of NTR and SHR Groups

**Figure 4:**
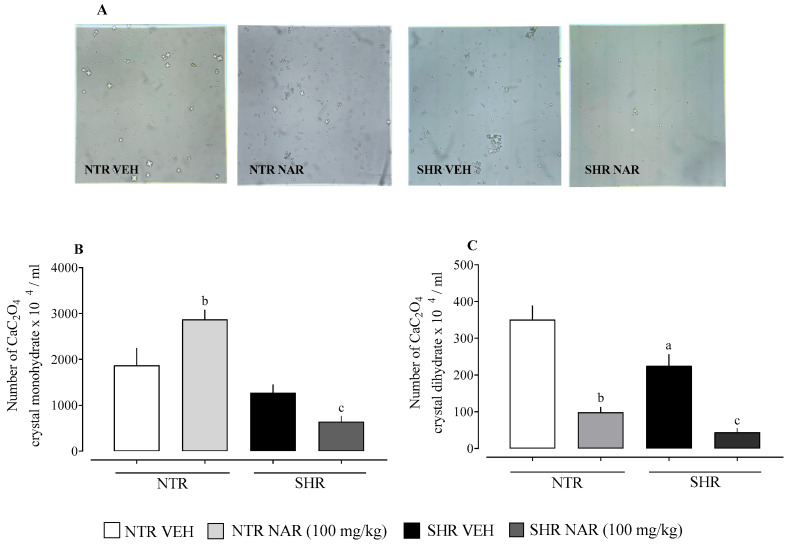
Inhibitory effects of naringenin on urinary stones induced by CaC_2_O_4_ precipitation: (**A**) illustrative image of crystals of each group at 400× magnification; (**B**) the number of CaOx monohydrate crystals; (**C**) the number of dihydrate crystals. ^a^ *p* < 0.05, comparison between NTR and SHR vehicles. ^b^ *p* < 0.05, comparison between the NTR vehicle and NTR NAR. ^c^ *p* < 0.05, comparison between SHR vehicle and SHR NAR.

The protective effect of naringenin on the formation of CaOx crystals in vitro was evaluated. Analysis of the results showed an increase in the formation of monohydrate crystals and a decrease in dihydrate crystals in the NTR NAR groups compared to the NTR VEH groups. In the SHR groups, there was a significant decrease in both monohydrate and dihydrate crystals (Figure 4).

### 2.5. Molecular Docking

While traditional factors such as urinary pH, calcium excretion, and fluid intake are well-known contributors to kidney stone formation, emerging evidence underscores the importance of specific enzymes and receptors in modulating urolithiasis-related pathways [18]. These molecular targets influence processes such as crystal nucleation, aggregation, and renal epithelial integrity.

**Figure 5:**
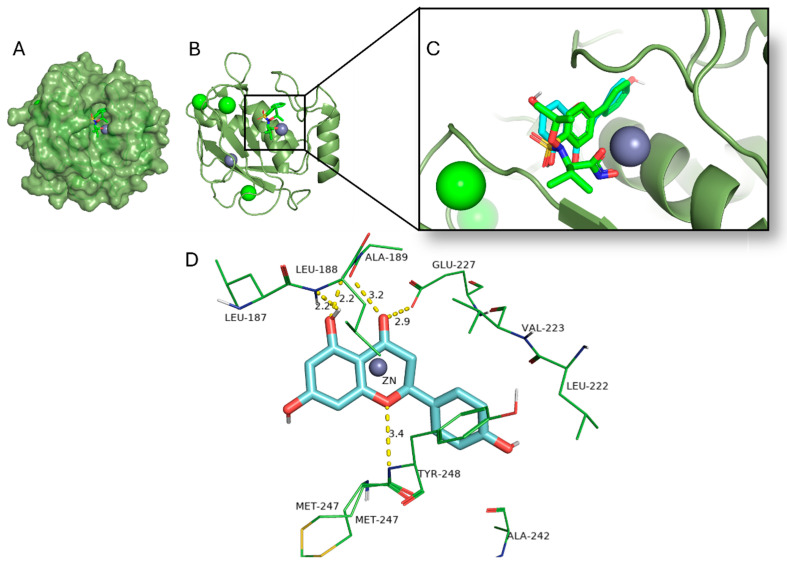
The MMP-9 catalytic domain surface (**A**); the ARP101 inhibitor binding site (**B**); comparison of ARP101 inhibitor biding pose (green) with naringenin binding pose (cyan) (**C**); naringenin and its predicted H-bonds (**D**).

The predicted binding affinities of naringenin to each selected protein target are presented in Table 1. The best predicted binding affinity was obtained for the MMP-9 (Figure 5), the SGLT 1 and 2 receptors, and the beta 2 adrenergic receptor.

The MMP-9 residues that most significantly contributed to the binding energy were identified as Leu222, Val223, Ala242, Met247, and Tyr248, facilitating the formation of a stable complex [19]. The selected PDB macromolecule was 4XCT, where the original ligand, a hydroxamate-based inhibitor ARP101, interacts with the protein through H-bonds with Leu188 and Ala189, as well as with the Zn^2+^ ion. Additionally, it engages in hydrophobic interactions with Ala189, His226, His230, Leu187, Val223, and Met247. Accordingly, naringenin interacts with the MMP-9 protein through H-bonds with Ala189, Leu188, Glu277 and Tyr248, and also forms hydrophobic interactions with Met247, Tyr248, Leu222, His226, and Val223.

The binding sites of SGLT1 and SGLT2 have unique structural and functional characteristics that influence their selectivity for substrates and inhibitors. SGLT2, which is mainly found in the kidney, contains a hydrophobic pocket created by residues like Tyr290, Trp291, and Phe453. This pocket is particularly suited for C-glucoside inhibitors such as empagliflozin and dapagliflozin, which interact through π-stacking and H-bonds with His80, Asn75, and Gln457. On the other hand, SGLT1, dominant in intestinal glucose uptake, has a larger binding cavity more accommodating to bulkier inhibitors like LX2761 [20,21,22].

In the PDB 7VSI protein, the ligand empagliflozin acts as an antagonist, forming H-bonds with the residues Asn75, Ser287, Lys321, Phe98, Glu99, and Gln457. It also engages in hydrophobic interactions with Tyr290, Leu84, Val95, and Phe453. Naringenin, on the other hand, interacts through H-bonds with Thr87, His80, Asn75, Tyr290, and Gln457, while also participating in hydrophobic interactions with Phe98, Phe453, and Val95, and a π–anion interaction with Glu99.

For the 7WMV PDB protein, the ligand LX2761, a high-affinity inhibitor, forms H-bonds with Asn78, His83, Asp454, His525, and Trp291. It also exhibits a π–cation interaction with His83 and hydrophobic interactions with Leu87, Ile98, Phe101, Leu274, and Phe453. Naringenin interacts through H-bonds with Gly82, Leu87, Trp291, and Gln457, π-stacking interactions with His83, and hydrophobic interactions with Tyr290, Phe101, Phe453, and Leu87.

The binding pocket of the beta-2 adrenergic receptor (β2AR) is located within the transmembrane (TM) domain, primarily involving TM3, TM5, TM6, and TM7. Key residues critical for ligand interactions include Asp113, which forms an ionic interaction with the protonated amine of catecholamine ligands, and Ser203, Ser204, and Ser207, which engage in hydrogen bonding with the catechol hydroxyl groups of endogenous agonists like epinephrine and norepinephrine [23,24]. Additionally, Tyr316 and Asn312 contribute to stabilizing ligand binding through hydrophobic and polar interactions, respectively 915. Antagonists such as alprenolol occupy the same orthosteric site but exhibit distinct interaction patterns. Unlike agonists, antagonists typically lack polar interactions with TM5 serines and instead form extensive hydrophobic contacts with residues such as Val117, Phe193, and Phe289, stabilizing the receptor in an inactive conformation [23,24].

The PDB 3NYA structure includes the ligand aprenolol, which forms H-bonds with Asn312, engages in π-stacking with Phe290, and exhibits hydrophobic interactions with Val117, Val114, Thr110, Trp109, Phe193, and Phe289. Naringenin, on the other hand, interacts with H-bonds with Asp113, Val114, Ser203, Asn293, and Tyr316, while also participating in π-stacking with Phe290 and hydrophobic interactions with Val117, Phe193, Thr110, and Tyr308.

## 3. Discussion

SAH is a health problem that affects millions of individuals worldwide and represents a significant risk factor for the development of cardiovascular and kidney diseases [25]. The kidneys play a key role in blood pressure control and regulation through blood volume and sodium excretion. A fundamental mechanism for maintaining electrolyte balance and blood pressure control is the RAAS [26]. Diuretics are drugs widely used in the treatment of cardiovascular and renal dysfunctions. Their main function is to increase the elimination of water and salt through the urine. There are five different types of diuretic, each of which works on a specific part of the kidney [27]. Even with the development of new drugs for hypertension, diuretics are still considered fundamental in the treatment, either alone or in combination with other drugs such as beta-blockers and those that act on the RAAS. Among the most commonly used to control blood pressure are loop diuretics, thiazide diuretics and potassium-sparing diuretics [28]. The biggest problem with diuretics is that they can cause electrolyte imbalances and other unwanted effects [29]. Therefore, it is essential to continuously seek therapeutic strategies targeting different components of this system to manage SAH and its complications.

Naringenin, a well-studied flavonoid found in citrus fruits, has demonstrated multiple therapeutic properties, including blood pressure regulation [17] and diuretic activity [16]. Building on these findings, the present study aimed to further explore the systemic effects of naringenin following repeated-dose administration, through the evaluation of cardiovascular and renal parameters not yet fully elucidated in the literature. Although numerous studies have explored the biological effects of naringenin, its role in modulating renal function remains underinvestigated. Understanding these renal effects may help clarify systemic actions attributed to naringenin, such as the regulation of blood pressure, which was also confirmed in the present study.

SHRs are a genetic model of hypertension development, created through the crossbreeding of isogenic strains. They have become the most widely used model in research involving cardiovascular disorders. The symptoms observed in this model are similar to those of SAH in humans. In addition to elevated blood pressure, SHRs also develop cardiac hypertrophy and other alterations commonly seen in hypertensive patients [30,31]. In addition, it is possible to observe renal alterations in SHR groups when compared to NTR groups, such as increased glomerular size, thickening of Bowman’s capsule and rupture of the mesangial space, in addition to progressive decline in renal function [32,33,34].

The results obtained confirm what has already been shown in the literature, that SHR groups have higher baseline blood pressure than NTR groups [17,31]. Continuing with the analyses, during treatment, it was possible to observe that daily exposure to naringenin proved to be crucial for the antihypertensive effect. This finding corroborates previous studies which suggest the therapeutic potential of naringenin in controlling blood pressure at doses of 50 and 100 mg/kg [15,17], although the exact mechanisms have yet to be fully elucidated. The reduction in HR observed in the NTR group treated with NAR may reflect a mild negative chronotropic effect, likely related to improved autonomic balance or reduced sympathetic activity under physiological conditions. In contrast, this effect was not seen in SHR, likely due to the persistent sympathetic overactivity typical of this model, which limits heart rate modulation. These findings suggest that NAR’s effects on HR are more evident in normotensive conditions and are less effective in the presence of chronic sympathetic dominance.

With regard to renal function, it was possible to observe a diuretic effect of naringenin in NTR and SHR groups, as well as a natriuretic effect in NTR groups. This result is of considerable importance since, by increasing the excretion of sodium and water by the kidneys, the volume of circulating blood is reduced, leading to a reduction in cardiac output and, consequently, blood pressure. The natriuretic effect is considered significant because sodium contributes to water retention in the body. Thus, regulating kidney function is of paramount importance for long-term blood pressure control [35,36]. The results of Vilhena da Silva et al. [16] corroborate our findings, indicating a diuretic effect of NAR in SHR groups.

Diuretics that modulate calcium are used strategically in the treatment of specific comorbidities, such as calcium-sparing diuretics in cases of osteoporosis and renal calcium [37,38,39,40,41]. Considering the modulation of renal and electrolyte function, the formation of CaOx crystals in urine samples from VEH and treated groups were evaluated. The results found by Vilhena da Silva et al. [16] corroborate the findings of this study, in which there was no significant decrease in the Ca^2+^ levels of the male SHR groups treated with NAR, but, on the other hand, there was a decrease in the formation of crystals in this same group. The gap in the literature does not allow us to compare the formation of CaOx crystals in NTR groups treated with NAR. However, this study indicates a significant correlation between the elimination of Ca^2+^ and the decrease in crystal formation in NTR groups [16].

The molecular docking profile of naringenin with a curated set of protein targets implicated in urolithiasis, renal electrolyte regulation, and vascular homeostasis was performed in this study. The docking analysis revealed that naringenin exhibited favorable binding affinities to several proteins, most notably MMP-9 (–10.4 kcal/mol), SGLT2 (–9.6 kcal/mol), SGLT1 (–9.4 kcal/mol), and Beta 2 adrenergic receptor (–9.2 kcal/mol), suggesting a multifaceted mechanism of action potentially relevant to nephroprotection.

Matrix metalloproteinases, particularly MMP-9, are zinc-dependent endopeptidases involved in extracellular matrix (ECM) remodeling, inflammation, and kidney injury, all critical events in the pathophysiology of urolithiasis [42]. The high binding affinity of naringenin to MMP-9, coupled with its ability to establish hydrogen bonds with key residues (Leu188, Ala189), indicates potential inhibitory activity, although naringenins phenolic groups lack strong Zn^2+^-chelating properties, which is important for inhibitor activity [19]. Previous studies have demonstrated that MMP-9 expression is upregulated in acute kidney injury, interstitial fibrosis, glomerulonephritis, and diabetic nephropathy [42]; therefore, MMP-9 inhibition by naringenin could attenuate tissue damage and prevent crystal nucleation, contributing to an anti-urolithiatic effect.

The sodium–glucose cotransporters SGLT1 and SGLT2 are primarily involved in glucose reabsorption in the small intestine and proximal tubule, respectively. Their inhibition not only reduces glycemic burden but also promotes osmotic diuresis and natriuresis which decreases the tubular sodium load, reduces glomerular hyperfiltration, and lower intraglomerular pressure, mechanisms with potential relevance for renal protection [43,44,45]. Naringenin’s binding to SGLT1 (−9.4 kcal/mol) and SGLT2 (−9.6 kcal/mol) indicates that it may modulate tubular glucose and sodium handling. These findings align with previous reports showing that naringenin enhances urinary volume and reduces urinary saturation of stone-forming solutes in rodent models [16,46]. Inhibition of SGLTs may thus contribute to dilution of lithogenic substances, an important preventive mechanism in urolithiasis.

The β2-adrenergic receptor (β2AR) plays a pivotal role in regulating renal perfusion, vascular tone, and smooth muscle relaxation. β2AR signaling mechanisms are integrated with multiple intracellular pathways within the kidney, playing a crucial role in regulating key physiological processes such as renal blood flow, electrolyte homeostasis, salt handling, glomerular filtration rate, and the modulation of renin secretion by juxtaglomerular cells [47]. Naringenin’s high affinity (−9.2 kcal/mol) and interaction with critical residues (e.g., Ser203, Asp113, Tyr316) suggest potential activity on β2AR, especially agonist, due to its interaction with the Ser203 residue, which could mediate vasorelaxant effects [23]. This is consistent with experimental findings that flavonoids, including naringenin, promote endothelium-dependent vasodilation via nitric oxide signaling pathway [48,49]. Also, in pathologic conditions like acute kidney injury, β2AR activation reduces inflammation by downregulating TNF-α and IL-6 via cAMP-PKA signaling [47]. Improved renal perfusion may indirectly reduce ischemic injury, inflammation, and stone-forming conditions.

Naringenin also demonstrated moderate to strong binding affinity with other urolithiasis and renal protection-related targets, including glycolate oxidase (−8.3 kcal/mol), a key enzyme in oxalate synthesis [50,51], and ACE (−7.9 kcal/mol), involved in the renin–angiotensin system [52]. Inhibition of glycolate oxidase could lower endogenous oxalate production, whereas ACE inhibition may mitigate proteinuria and renal injury, contributing to a nephroprotective phenotype [50,51,52,53].

Therefore, it is suggested that the possible mechanism by which naringenin reduces blood pressure is by modulating renal function, promoting diuresis and natriuresis, possibly targeting the SGLT1 and SGLT2 receptors, as observed through docking. In addition, its inhibitory effect on the formation of calcium oxalate crystals is possibly related to a decrease in Ca^2+^ excretion by the urine and, additionally, to a reduction in inflammation and modulation of renal irrigation, which, according to our docking results, may be linked to interactions with the MMP-9 enzyme and the β2AR receptor.

Although this study did not include hormonal analysis or histopathological evaluation, the findings provide important evidence of the beneficial effects of naringenin on blood pressure regulation, diuresis, and reduction of calcium oxalate crystal formation. Future studies should explore different dosing protocols, longer treatment durations, and include additional parameters such as kidney injury biomarkers and molecular interaction assays to further clarify the underlying mechanisms.

## 4. Materials and Methods

### 4.1. Drugs

Naringenin, with 98% purity, was sourced from Sigma–Aldrich (St. Louis, MO, USA). All other reagents employed were of analytical grade and acquired from Merck (Darmstadt, Germany).

### 4.2. Animals

Male Wistar rats, normotensive (NTR) and spontaneously hypertensive (SHR), aged between 3 and 4 months, 250–300 g weight, were obtained from the UNIVALI Central Vivarium. The animals were housed under controlled environmental conditions (22 ± 2 °C), with a 12 h light/dark cycle, and had free access to standard chow and water. All experimental procedures were approved by the Institutional Ethics Committee of Universidade do Vale do Itajaí (protocol code 017/22 and 005/23, date of approval 1 June 2022 and 13 April 2023, respectively).

### 4.3. Treatments

The treatment was carried out over 9 days, administered orally (by gavage), once a day in the morning, after fasting for 2 h. The NTR and SHR VEH groups received 0.9% saline solution (1 mL/ 100 g body weight), while the NTR and SHR NAR groups were treated with naringenin (100 mg/kg). The dose of 100 mg/kg of NAR was selected based on previous studies demonstrating its efficacy and safety to produce significant pharmacological effects, including antioxidant and antihypertensive activities, without causing toxicity [16,17].

### 4.4. Blood Pressure Measurement via Tail-Cuff Plethysmography

This study involved both control and experimental groups. Measurements were taken every two days, with diuresis occurring during this period. The initial measurement was conducted before the treatment began, and subsequent measurements were taken two hours post-treatment to assess the compound’s impact before and after administration. Systolic blood pressure (SBP), diastolic blood pressure (DBP), mean arterial pressure (MAP), and heart rate (HR) were measured using plethysmography (Serial number: 007006; Bonther, Ribeirão Preto, SP, Brazil). To minimize stress from the restraint needed for blood pressure measurement, both the vehicle and treated groups were acclimated to the equipment a week prior to the first measurement. After acclimatization in a room heated to 28–30 °C, the animals were placed in acrylic containment tubes on a heated plate. A previously calibrated transducer was attached to a sphygmomanometer on the animal’s tail, which featured an automated inflation system linked to a data capture and conversion system, connected to a computer with specific data acquisition software (Tail Plethysmography. Ink, Bonther, Ribeirão Preto, SP, Brazil).

### 4.5. Determination of Diuretic Activity and Urine Analysis

NTR and SHR treated groups, after 2 h fasting with free access to water, received an oral load, by gavage, of naringenin (100 mg/kg) and the vehicle groups received saline solution (0.9%). Immediately after treatment, the rats were placed individually in metabolic cages, where they were kept for 8 h, repeating the process for five days, with a one-day break. The urine output of each rat was collected daily, and the volume was measured at the end of the five days. Urine volume was standardized and expressed as mL per 100 g of body weight. The urinary pH was determined using a digital pH meter (mPA-210; MS Tecnopon, Piracicaba, SP, Brazil). Sodium (Na^+^) and potassium (K^+^) excretions were quantified with a flame photometer (BFC-300; Benfer, São Paulo, SP, Brazil). The concentrations of chloride (Cl^−^), calcium (Ca^2+^), urea, and creatinine in the urine samples were measured using commercial colorimetric assay kits (Bioclin, Belo Horizonte, MG, Brazil).

### 4.6. Induction of Calcium Oxalate (CaOx) Precipitation and Crystallization

The method used for this protocol was adapted from Yousefi Ghale-Salimi et al. (2018) [54]. Urine samples were collected during diuresis from male NTR and SHR with naringenin treatment as described above. In total, 80 μL of 0.1 M sodium oxalate was added to the urine samples (500 μL) to induce CaOx precipitation. The samples were incubated at 37 °C for 60 min and subsequently analyzed using a Neubauer chamber by observing four randomly selected fields. Crystal morphology was identified and categorized as either calcium oxalate monohydrate or calcium oxalate dihydrate forms.

### 4.7. Molecular Docking 

Molecular docking for naringenin was conducted using crystal structures of selected enzymes, which were obtained from the Protein Data Bank (PDB) [55]. The proteins were prepared with the AutoDock Tools (ADT 1.5.7) program [56], where co-crystal ligands, water, and ions were removed, hydrogens were added, and Gasteiger charges were calculated. The 3D structure of naringenin, retrieved in mol2 format from PubChem (2025), was also prepared for docking using the ADT 1.5.7 program. Docking was executed with AutoDock Vina [57], using a grid box centered on the active site with dimensions sufficient to accommodate ligand flexibility. Validation of the docking protocol was performed by redocking the native ligand and calculating RMSD (<2 Å considered acceptable). The potential interactions and 3D images were generated using PyMOL 3.0.3 (Schrodinger LLC, New York, NY, USA, 2015), while 2D images were produced with the BIOVIA Discovery Studio program.

The selection of enzymes was predicated on their potential involvement in various physiological processes, such as in the pathogenesis of urolithiasis directly, or indirectly, as diuresis and vascular effects. The enzymes comprise glycolate oxidase (PDB ID: 2RDT), calcium-sensing receptor within the Venus flytrap (VFT) domain (PDB ID: 5FBK), calcium-sensing receptor in the 7-transmembrane (7TM) domain (PDB ID: 7DD7), matrix metalloproteinase (MMP)-1 (PDB ID: 3SHI), MMP-2 (PDB ID: 1CK7), MMP-9 (PDB ID: 4XCT), phosphoethanolamine cytidylyltransferase (PDB ID: 3ELB), epithelial sodium channel (PDB ID: 6BQN), and Na–K–Cl cotransporter (NKCC) 1 (PDB ID: 7SFL) for the urolithiasis pathogenesis. Additionally, enzymes potentially implicated in diuresis, excretion of electrolyte-free water or involved in vascular effects were also selected, including angiotensin receptor type 1 (PDB ID: 4ZUD), angiotensin receptor type 2 (PDB ID: 5UNF), sodium–glucose cotransporter (SGLT) 1 (PDB ID: 7WMV), SGLT 2 (PDB ID: 7VSI), vasopressin receptor (PDB ID: 7DW9), vasopressin V2 receptor (PDB ID: 7BB7), the angiotensin-converting enzyme (ACE) (PBD ID: 1O86), renin (2V0Z), and the beta 2 adrenergic receptor (PDB ID: 3NYA).

### 4.8. Statistical Analysis

Data are presented as mean ± standard error of the mean (SEM), with group sizes ranging from 6 to 8 animals. Statistical comparisons were performed using two-way ANOVA followed by Bonferroni’s post hoc test (Figure 1) or one-way ANOVA followed by Dunnett’s post hoc test (Figure 2, Figure 3 and Figure 4). Analyses were conducted using GraphPad Prism version 8.0.1 (GraphPad Software, La Jolla, CA, USA). Differences were considered statistically significant when *p* < 0.05.

## 5. Conclusions

Taken together, the results of this study suggest that repeated treatment with NAR exerts multiple beneficial effects on cardiovascular and renal physiology. NAR demonstrated a significant antihypertensive effect in SHR, likely mediated by improved vascular homeostasis and natriuretic mechanisms. Additionally, it promoted a marked diuretic response in both NTR and hypertensive animals, accompanied by enhanced natriuresis in NTR, suggesting potential modulation of renal tubular sodium handling. Importantly, NAR also demonstrated the capacity to inhibit calcium oxalate crystallization in vitro, indicating a possible protective role against the development of nephrolithiasis. These combined actions highlight NAR as a multifunctional bioactive compound with therapeutic potential for managing hypertension, fluid–electrolyte imbalances, and renal stone formation. Furthermore, molecular docking analysis revealed potential interaction with key renal and cardiovascular targets, which opens new perspectives for mechanistic exploration. Nevertheless, further studies are essential to clarify the precise molecular pathways involved, assess the efficacy in chronic models, evaluate pharmacokinetics, bioavailability, and safety, and eventually validate its translational potential for clinical application.

## Figures and Tables

**Table 1:** Predicted molecular docking binding affinity of naringenin with selected enzymes possibly involved in urolithiasis pathogenesis.

Enzyme	PDB ID	Naringenin
Angiotensin Type 2	5UNF	−8.4
Calcium-sensing receptor EX	5FBK	−8.1
Calcium-sensing receptor 7TMD	7DD7	−8.5
Glycolate Oxidase	2RDT	−8.3
MMP-1	3SHI	−7.5
MMP-2	1CK7	−7.6
MMP-9	4XCT	−10.4
Sodium–Glucose Cotransporter 1	7WMV	−9.4
Sodium–Glucose Cotransporter 2	7VSI	−9.6
Vasopressin	7DW9	−7.9
Vasopressin V2	7BB7	−6.6
Phosphoethanolamine Cytidylyltransferase	3ELB	−8.2
ACE	1O86	−7.9
Renin	2V0Z	−7.9
Beta 2 adrenergic receptor	3NYA	−9.2
ENaC	6BQN	−7.8
NKCC1	7SFL	−6.6

PDB ID: Protein Data Bank identifier for the 3D structure of the protein; 7TMD: seven transmembrane domain; MMP: matrix metalloproteinase enzyme; ACE: angiotensin-converting enzyme; ENaC: epithelial sodium channel; NKCC1: sodium–potassium–chloride cotransporter 1.

## Data Availability

The original contributions presented in the study are included in the article, further inquiries can be directed to the corresponding author.
